# Revisiting kinorhynch segmentation: variation of segmental patterns in the nervous system of three aberrant species

**DOI:** 10.1186/s12983-021-00438-5

**Published:** 2021-10-21

**Authors:** Maria Herranz, Taeseo Park, Maikon Di Domenico, Brian S. Leander, Martin V. Sørensen, Katrine Worsaae

**Affiliations:** 1 Department of Biology, University of Copenhagen, Copenhagen, Denmark; 2 Natural History Museum of Denmark, University of Copenhagen, Copenhagen, Denmark; 3 National Institute of Biological Resources, Incheon, South Korea; 4 Centro de Estudos do Mar, Universidade Federal do Paraná, Pontal do Paraná, Brazil; 5 Departments of Zoology and Botany, University of British Columbia, Vancouver, Canada

**Keywords:** Acetylated tubulin, Confocal laser scanning microscopy, FMRFamide, Kinorhyncha, Meiofauna, Nervous system, Serotonin, Mismatch

## Abstract

**Background:**

Kinorhynch segmentation differs from the patterns found in Chordata, Arthropoda and Annelida which have coeloms and circulatory systems. Due to these differences and their obsolete status as ‘Aschelminthes’, the microscopic kinorhynchs are often not acknowledged as segmented bilaterians. Yet, morphological studies have shown a conserved segmental arrangement of ectodermal and mesodermal organ systems with spatial correspondence along the anterior-posterior axis. However, a few aberrant kinorhynch lineages present a worm-like body plan with thin cuticle and less distinct segmentation, and thus their study may aid to shed new light on the evolution of segmental patterns within Kinorhyncha.

**Results:**

Here we found the nervous system in the aberrant *Cateria styx* and *Franciscideres kalenesos* to be clearly segmental, and similar to those of non-aberrant kinorhynchs; hereby not mirroring their otherwise aberrant and posteriorly shifted myoanatomy. In *Zelinkaderes yong*, however, the segmental arrangement of the nervous system is also shifted posteriorly and misaligned with respect to the cuticular segmentation.

**Conclusions:**

The morphological disparity together with the distant phylogenetic positions of *F. kalenesos*, *C. styx* and *Z. yong* support a convergent origin of aberrant appearances and segmental mismatches within Kinorhyncha.

## Background

Segmentation is one of the most ambiguous and discussed concepts among evolutionary biologists. Nonetheless, some of the generally accepted conditions for considering an organism segmented are: showing a spatial alignment of serially repeated elements originating from different germ layers, (e.g., mesoderm and ectoderm derivatives); temporal coordination of the development of these elements; sequential anterior-posterior segment formation; and serial expression of segment polarity genes [1–4]. Based on these premises “true” segmentation has only been accredited to arthropods, annelids, and chordates, although debated for other animal groups (summarized in [5]). Due to their distant phylogenetic relationship, this segmentation is assumed to have evolved three times independently [3]. However, an additional and often overlooked group of segmented animals exists, namely Kinorhyncha or mud dragons. These are microscopic marine invertebrates inhabiting most benthic environments across the globe. Adult kinorhynchs exhibit a conserved body plan with a radial head bearing a terminal mouth, a neck, and a trunk composed of eleven articulated segments [6, 7]. Within the trunk, the musculature, nervous system, epidermal glands and sensory organs are segmentally arranged [6–10]. Yet, the development of the segments pre- and post-hatching is poorly studied (e.g., [11–13] summarized in [7]). It is therefore still uncertain whether there is a temporal coordination in the development of the different organ systems or how segment polarity genes are expressed in Kinorhyncha.

Traditionally, kinorhynchs were grouped with non-segmented bilaterians (Rotifera, Gastrotricha, Nematoda, Nematomorpha and Priapulida among others) in the heterogeneous and now obsolete group Aschelminthes, also referred to as Nemathelminthes, based on the absence of a true coelom [14–16]. Thus, the kinorhynch body plan was traditionally never considered segmented but metameric [2, 5]. Hyman [14] considered kinorhynchs to be “superficially segmented”, equivalent to the annulated trunks observed in nematodes and priapulids. Subsequently, many studies followed Hyman’s 1951 interpretation of kinorhynchs being “superficially segmented”, “pseudosegmented” or with a body plan of uncertain condition [17–21] usually naming segments as “zonites” in order to stress their non-segmental character [22], whereas other books and studies from the same period considered them as a segmented animal group (e.g., [23–25]). As the general opinion gradually changed towards favouring the Ecdysozoa over the Articulata hypothesis, it seems like most studies also tended to accept kinorhynchs as segmented, although still maintaining the term “zonite”. However, no new morphological evidence was produced to support this. Thus, interpretations of kinorhynchs as unsegmented, “superficially segmented”, “pseudo-segmented” or “truly segmented” were mostly based on speculations, old ultrastructural data from a restricted number of ‘model species’, and ‘whatever fits the current tree’, until more recent studies finally produced new evidence using immunohistochemistry (e.g., [8–10, 26–30]).

Kinorhynchs are now reliably nested within the clade of moulting bilaterians, known as Ecdysozoa [31], though herein only distantly related to the segmented Arthropoda. Although the relationships within Ecdysozoa are still debated (see Giribet and Edgecombe [32] and references herein), morphology consistently supports that Kinorhyncha and the non-segmented Priapulida and Loricifera form the clade Scalidophora (animals with radial heads bearing scalids). Together with the non-segmented phyla Nematoda and Nematomorpha, they form the sister clade of Panarthropoda called Cycloneuralia (referring to the presence of a ring-shaped brain around the pharynx) [33–35]. Within panarthropods only arthropods are fully segmented, although the closely related onychophorans and tardigrades show serial pattering in some organ systems (e.g., [36, 37]). With kinorhynchs being closely related to unsegmented vermiform lineages and only distantly related to arthropods, the study of kinorhynch segmentation becomes pivotal to understanding the evolution of segmentation in Ecdysozoa [8, 9].

Over the last two decades, an increasing number of morphological studies based on immunohistochemistry, confocal laser microscopy (CLSM) and three-dimensional rendering have addressed kinorhynch internal anatomy in a variety of genera complementing traditional transmission electron microscopy-based investigations (e.g., [9, 10, 28–30]). Comprehensive myoanatomical studies in kinorhynchs have so far been achieved in representatives of eight genera (*Antygomonas*, *Cateria*, *Echinoderes*, *Franciscideres, Dracoderes*, *Pycnophyes*, *Setaphyes* and *Zelinkaderes*) where all of them show a segmental pattern of at least dorsoventral and longitudinal muscle sets [8, 10, 27, 29, 30, 38]. Neuroanatomical studies using immunolabeling have been carried out for five genera (*Antygomonas*, *Echinoderes*, *Dracoderes*, *Pycnophyes* and *Setaphyes*) [9, 10, 26, 30]. These studies show a similar nervous system architecture that mirrors the anterior-posterior pattern of repeated units shown by the tegumental plates and the musculature in most of the segments [8–10, 30, 39].

Aberrant kinorhynchs are distantly related (cyclorhagid and allomalorhagid) genera (see Sørensen et al. [40]) that share several modifications from the typical kinorhynch body architecture (see [27, 41]) most conspicuously having a more elongated trunk, a flexible thin cuticle that folds during movement, and less differentiated trunk segments. Studies of these external divergences and how they relate to the organization of the subjacent organ systems will offer a deeper insight into the evolution of segmentation in kinorhynchs. Myoanatomical studies on three aberrant genera and species *Cateria styx* Gerlach, 1956; *Franciscideres kalenesos* Dal Zotto, Di Domenico, Garraffoni & Sørensen, 2013; and *Zelinkaderes yong* Altenburger, Rho, Chang & Sørensen, 2015 have recently been carried out by Herranz et al. [27]. Results showed that the trunk muscles follow a segmental arrangement in the three studied species; however, the longitudinal muscles showed a posterior shift in their position in respect to the segmental organization of the tegumental plates. This was interpreted to be a consequence of the absence of cuticular thickenings (pachycycli) normally used for muscle attachment in non-aberrant kinorhynchs. Additionally, the elongation of the trunk, the thin cuticle, and less distinct external segmentation was interpreted as an adaptation to the interstitial environment [27].

Here, we study the neuroanatomy of three aberrant kinorhynchs with an elongated, worm-like body plan and less distinct segmentation, *Cateria styx*, *Franciscideres kalenesos* and *Zelinkaderes yong*. The myoanatomy of these species was reconstructed by Herranz et al. [27] and the patterns will be compared to their neuroanatomy here examined through immunohistochemistry and CLSM with a standard selection of molecular markers (antibodies against acetylated α-tubulin, FMRFamides, and serotonin). Our aims are furthermore to investigate: (1) whether the nervous system of aberrant kinorhynchs shows a segmental pattern congruent with the external segmentation as in remaining kinorhynchs, (2) or whether there is any posterior shift of the neural components along an anterior-posterior axis mirroring their aberrant myoanatomy, (3) revisit and discuss the spatial alignment of segmental patterns in the cuticle, nervous system and musculature across all studied kinorhynchs, (4) compare and discuss segmentation patterning between kinorhynchs and other segmented phyla.

## Results

**Fig. 1:**
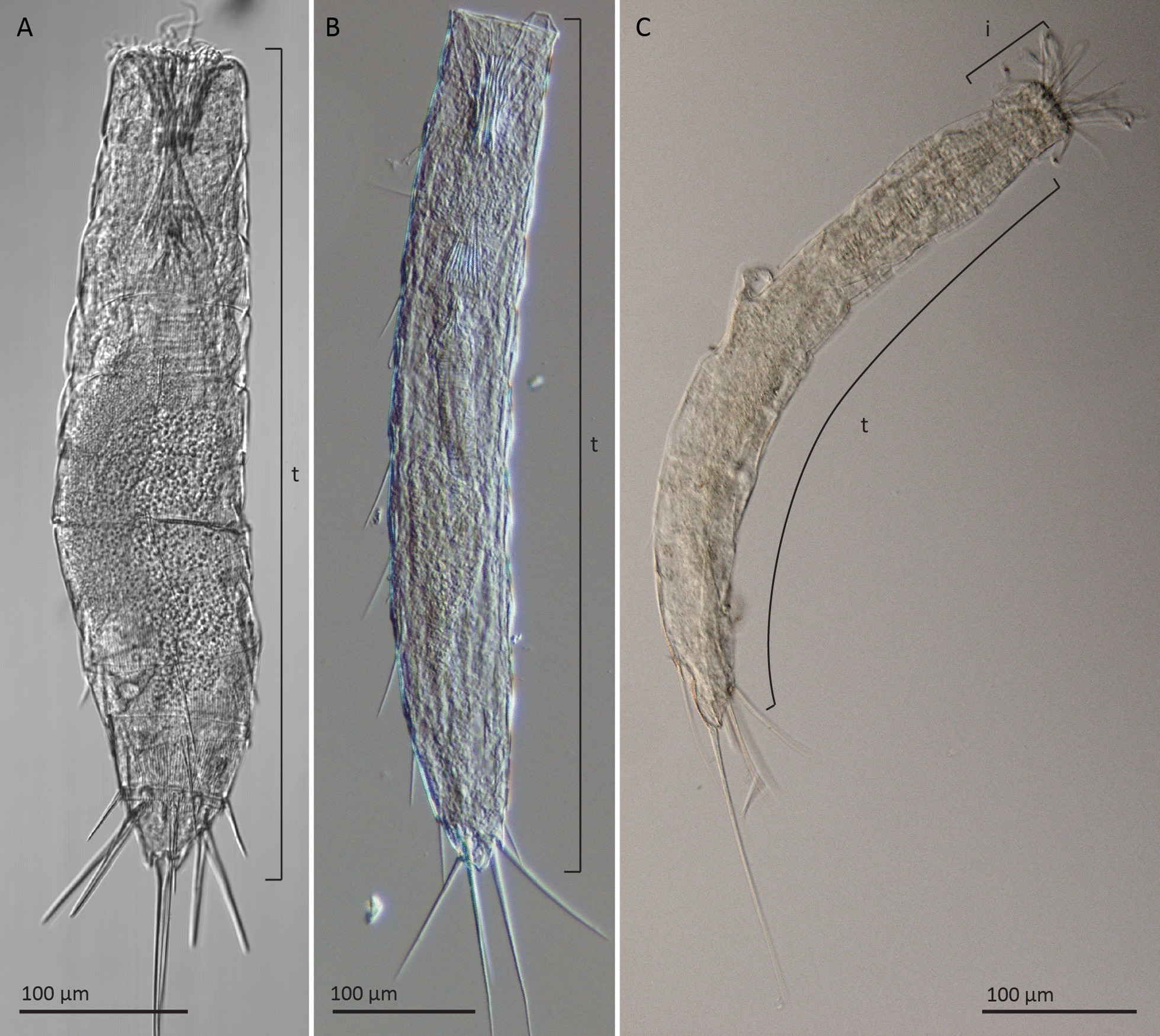
Differential interference contrast (DIC) micrographs showing the external anatomy of the three studied species. **A** *Zelinkaderes yong*. **B** *Franciscideres kalenesos.* **C** *Cateria styx*

The three studied species (*F. kalenesos*, *C. styx* and *Z. yong*) are representatives of three aberrant kinorhynch genera (Fig. 1). Aberrant kinorhynchs show distinct morphological characters that differ from the typical kinorhynch morphology such as: modified introverts with fewer spinoscalids and very elongated tentacle-like primary spinoscalids; weakly developed or, in some cases, absent neck; elongated trunk with thin, flexible cuticle and less distinct segmentation. For additional information on aberrant kinorhynch species see Herranz et al. [27]. For details on the phylogenetic position of the selected aberrant kinorhynchs see Sørensen et al. [40]. Comprehensive descriptions of cuticular morphology of *F. kalenesos* can be found in Dal Zotto et al. [42] and Rucci et al. [43]; of *C. styx* in Higgins [44], Neuhaus and Kegel [45] and Herranz et al. [41], of *Z. yong* in Altenburger et al. [46].

Due to the numerous similarities found in the nervous system architecture in the three studied species, a detailed neuroanatomical description is only provided for *F. kalenesos* while differences to this are given for *C. styx* and *Z. yong*. Abbreviations: i, introvert; t, trunk.

### Neuroanatomy of *F. kalenesos*

#### Acetylated α-tubulin-like immunoreactivity

**Fig. 2:**
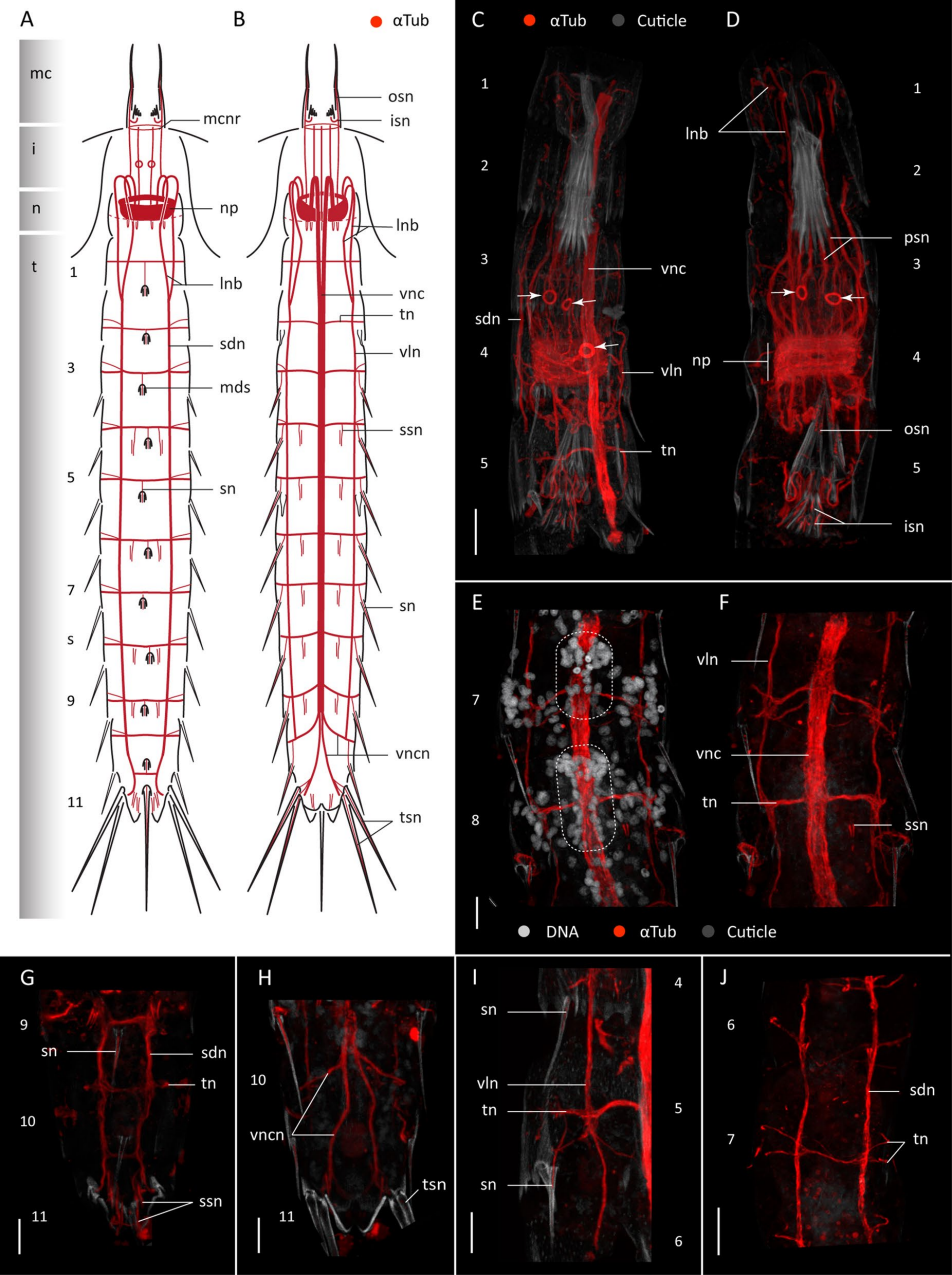
Acetylated α-tub-LIR in the nervous system of *Franciscideres kalenesos*. Anterior is up in all panels. **A**, **B**. Schematic representation in dorsal (**A**) and ventral (**B**) view. Grey shading on the left side marks the different body regions. For clarity, innervation of the introvert scalids and the ring-like structures associated to the midventral part of the brain neuropil in (**B**) have been omitted. Only the base of each of middorsal spine is marked in (**A**). **C–J**. confocal Z-stack projections of specimens co-labelled with acetylated α-tubulin (**C–J**) and DAPI (**E**). Autofluorescence of the cuticle was kept as guidance in (**C–J**). Colour legend in (**C**) applies to panels (**C**, **D, G–J**). **C** Segments 1-5 ventrolateral view, introvert retracted. **D** Same specimen as in (**C**) in dorsal view. Arrowheads mark the position of the α-tub-LIR ring-like structures associated with the introvert. **E**, **F** Detail of segments 7-8 ventral view, dotted areas mark the position of the ganglia in the ventral nerve cord. **G** Segments 9-11 dorsal view. **H** Segments 10-11 ventral view. **I** Detail of segments 4-5, right side. Note the innervation of the lateroventral spines of segments 4-5. **J** Segments 6-7, dorsal view. Scale bars: 20 μm (**C**, **D, J**); 10 μm (**E–I**). Abbreviations: i, introvert; isn, inner oral styles neurite; lnb, longitudinal neurite bundle; mc, mouth cone; mcnr, mouth cone nerve ring; mds, middorsal spine; n, neck; np, neuropil; osn, outer oral style neurite; psn, primary spinoscalid neurite bundle; sdn, subdorsal nerve; sn, spine neurite; ssn, sensory spot neurite; t, trunk; tn, transverse neurite bundle; tsn, lateroterminal spine neurite; vln, ventrolateral nerve; vnc, ventral nerve cord; vncn, ventral nerve cord neurite bundle. Numbers refer to segments

Acetylated α-tubulin-like immunoreactivity (α-tub-LIR) was studied in twenty specimens of *F. kalenesos*. α -tub-LIR is consistent in all studied specimens showing a circumpharyngeal brain neuropil (np), of ca. 15 μm width that narrows on the ventral side, composed of multiple densely arranged transverse neurites (Fig. 2A–D). The position of the brain varies depending on the level of introvert retraction, however when the introvert is everted the brain is located inside the neck or the first trunk segments.

From the anterior part of the brain neuropil ten longitudinal bundles (lnb) arise radially, extend anteriorly and bend 180° towards the body wall before extending posteriorly along the trunk (Fig. 2A–D). Of the ten neurite bundles the two ventromedial ones are the most prominent; they fuse in segment 1 forming the ventral nerve cord (vnc) (Fig. 2B, C). The remaining eight neurite bundles also fuse two by two at the level of segments 1-2, forming two ventrolateral and two subdorsal nerves (Fig. 2A, B). The ventral nerve cord extends in a midventral position along the trunk reaching the posterior end of segment 9, where it splits into four neurite bundles (vncn), two to the left and two to the right (Fig. 2B, C, E, F, H). Two of the ventral nerve cord neurite bundles extend laterally towards segment 10 and two towards segment 11 encircling each segment and connecting to the subdorsal nerves (Fig. 2A, B, G, H). The two ventrolateral nerves (vln) extend from segment 2 to segment 10 and the two subdorsal nerves (sdn) from segment 2 to segment 11 (Fig. 2A, B, G, H). All longitudinal cords are connected through a bundle of transverse ring neurites (tn). These neurite bundles originate from left and right sides of the ventral nerve cord and encircle segments 1-9 and the neck (Fig. 2A, B, E, F); the α-tub-LIR in the neck was very weak though. The transverse neurite bundles seem to split from ventrolateral to sublateral positions of most segments, yet the pattern is not consistent in every segment (Fig. 2J). Some segments also show additional thin transverse neurites in ventromedial positions (Fig. 2F).

Out of the five nerve cords the ventral nerve cord is the only one associated with paired clusters of nuclei. Nuclei are situated along the ventral nerve cord in the anterior half of each segment, being more numerous and densely clustered anterior to the arising point of the transverse neurite bundles (Fig. 2E, F). These paired clusters are interpreted as ganglia, present in segments 1-9; however, in segment 1 the nuclei form a unique dense cluster that seems to extend along the entire segment length. Whether this is a single large ganglion or two fused ganglia needs to be determined with ultrastructural studies. The intersegmental region of the ventral nerve cord does otherwise not have nuclei associated (Fig. 2E).

**Fig. 3:**
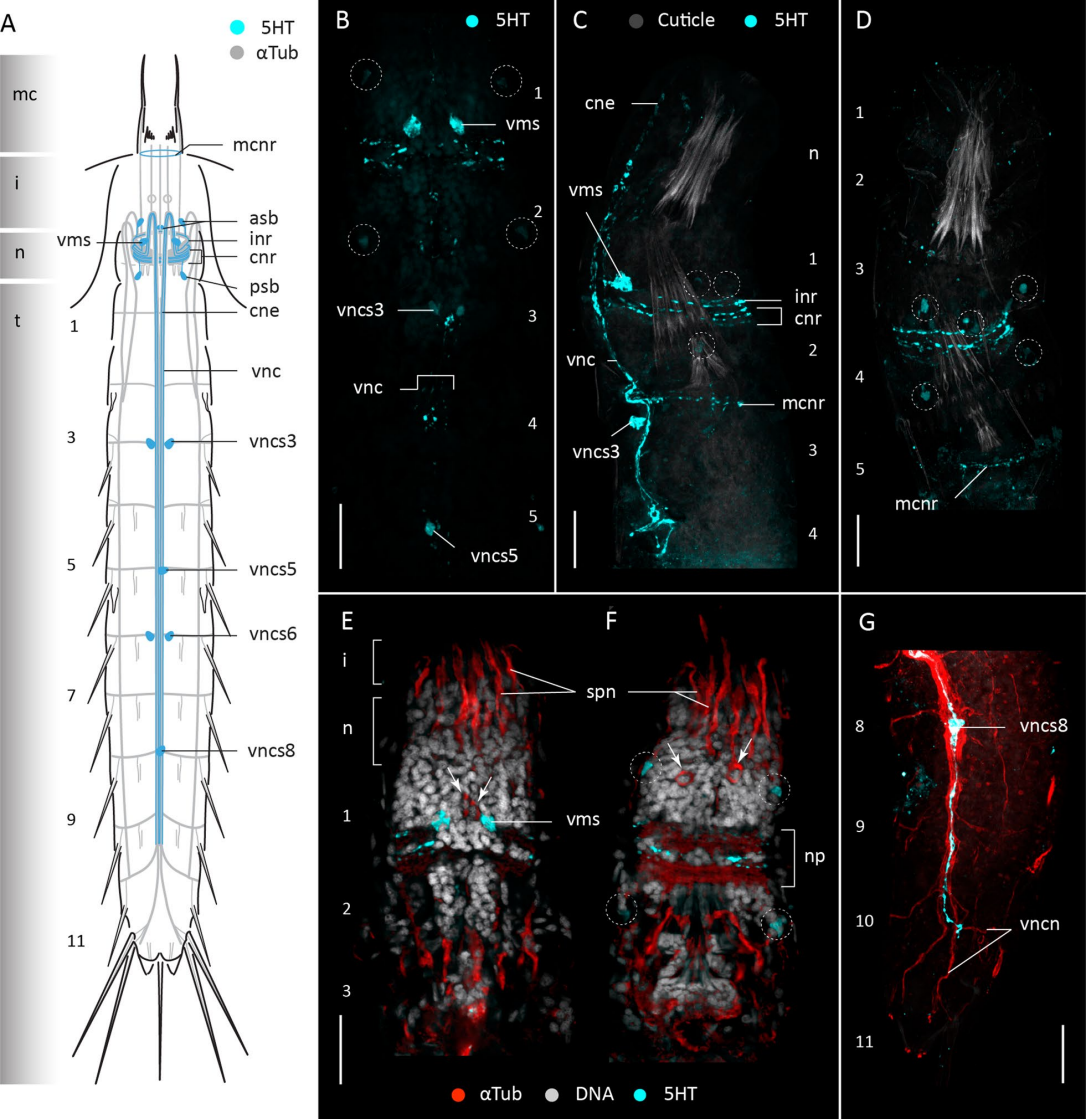
Serotonin-LIR in the nervous system of *Franciscideres kalenesos*. Anterior is up in all panels. **A** Schematic representation, ventral view. Grey shading on the left side marks the different body regions. **B–G** Confocal Z-stack projections of specimens co-labelled with 5HT (**B–G**), acetylated α-tubulin (**E–G**) and DAPI (**E**, **F**). Autofluorescence of the cuticle was kept as guidance in (**C**, **D**). Colour legend in (**C**) applies to (**D**), colour legend in (**E**, **F**) also applies to (**G**). **B** Detail of brain neuropil, ventral nerve cord and associated somata, ventral view. **C** Detail of brain neuropil, ventral nerve cord and associated somata, lateral view. **D** Detail of brain neuropil and associated somata, dorsal view. **E** Introvert, neck and segments 1-3 ventral view. **F** same specimen as in (**E**), dorsal view. Arrows mark the position of the circular α-tub-LIR structures (2 ventral in **E** and 2 dorsal in **F**). **G** segments 8-11, ventrolateral view.Dashed circles mark the position of the 5HT-LIR somata associated to the brain in (**B–F**). Scale bars: 20 μm. Abbreviations: asb, anterior somata of the brain; cne, convergent neurite; cnr, 5HT-LIR complete nerve ring; i, introvert; inr, 5HT-LIR incomplete nerve ring; mcnr, mouth cone nerve ring; n, neck; np, neuropil; psb, posterior somata of the brain; spn, spinoscalid neurite; t, trunk; vms, ventromedial somata of the brain; vnc, ventral nerve cord; vncs, ventral nerve cord somata; vncn, ventral nerve cord neurite bundle. Numbers refer to segments

Most cuticular structures such as introvert spinoscalids, mouth cone styles, trunk spines and sensory spots show intrinsic α-tub-LIR. Every introvert spinoscalid has a neurite or neurite bundle arising from the tip and connecting to the anterior part of the neuropil, except for the primary spinoscalids (psp) which show two prominent neurite bundles each (psn) (Figs. 2D, 3E, F). Inner and outer mouth cone oral styles, show neurites (isn, osn respectively) that arise in the tip of each style and bent 180° to connect to the posterior end of the neuropil (Fig. 2D). Inner and outer oral style neurites are encircled by a circular neurite bundle (mcnr) situated at the base of the inner oral styles. All trunk acicular spines (lateroventral and middorsal) show neurites (sn) originating in the tip of each spine, extending along its length and connecting to the transverse neurite of the corresponding segment (Fig. 2I). Lateral terminal spines and lateral terminal accessory spines of segment 11 also have neurites (tsn) that extend from the tip of each spine and connect to the ventral nerve cord neurites of this segment (Fig. 2A, B). Each sensory spot of the trunk shows two short parallel neurites (ssn), one of them extending from the external pore towards the transverse neurite bundle of the same segment (Fig. 2F–H).

Two pairs of α-tub-LIR ring-like structures (marked with arrowheads in Fig. 2C, D) were found in the dorsal part of the introvert, anterior to the brain neuropil, and ventromedially at the level of the brain neuropil (Fig. 2C, D). Due to the position of the rings, which overlap with the multiple neurite bundles emerging from the spinoscalids, it is not possible to see if there is any connection from the rings to the brain neuropil or the introvert. Specimens co-labelled with DAPI show that each of the ring-like α-tub-LIR structures surrounds a single cell nucleus. The two dorsal cells are regarded as part of the anterior brain region and the two ventral cells seem to be either part of the anterior brain region or the neuropil (Fig. 3E, F).

Non-neuro-specific α-tub-LIR was detected laterodorsally on segments 8-9 congruent with the position of the two nephridia.

#### Serotonin-like immunoreactivity

Serotonin-like immunoreactivity (5HT-LIR) was studied in sixteen specimens of *F. kalenesos*. All specimens show a similar 5HT-LIR present in the brain neuropil, at the base of the mouth cone and in the ventral nerve cord (Fig. 3). Within the neuropil, at least four to five serotonin immunoreactive rings can be detected; of these, the first three rings are ventrally incomplete (inr). The first ring extends from a pair of big ventromedial somata (vms). Associated somata of the second and third rings are not identified, but their two pairs of neurites extend anteriorly before bending 180 degrees and continuing posteriorly, first via the ventral convergent neurites (cne) and then via the fused ventral cord (vnc), until segment 10 (Fig. 3A–F). Remaining neuropil rings are complete (cnr) and are located posterior to the incomplete rings (Fig. 3A–C). Additionally, 5HT-LIR neurites are present forming a ring at the base of the mouth cone within the mouth cone nerve ring (mcnr) (Fig. 3A, C, D). Serotonin immunoreactive somata are associated with the anterior and posterior brain regions, as well as the ventral nerve cord. Within the anterior brain region, besides the ventromedial somata, there are two additional pairs of somata in laterodorsal and middorsal positions (asb) that connect with the neuropil (Fig. 3A–E). Within the posterior brain region there is one pair of laterodorsal somata (psb) connected to the neuropil (Fig. 3A–F). Along the ventral cord, paired somata (vncs) are present in the ganglia of segments 3 and 6, and unpaired median somata in the ganglia of segments 5 and 8 (Fig. 3A–C, G).

#### FMRFamide-like immunoreactivity

**Fig. 4:**
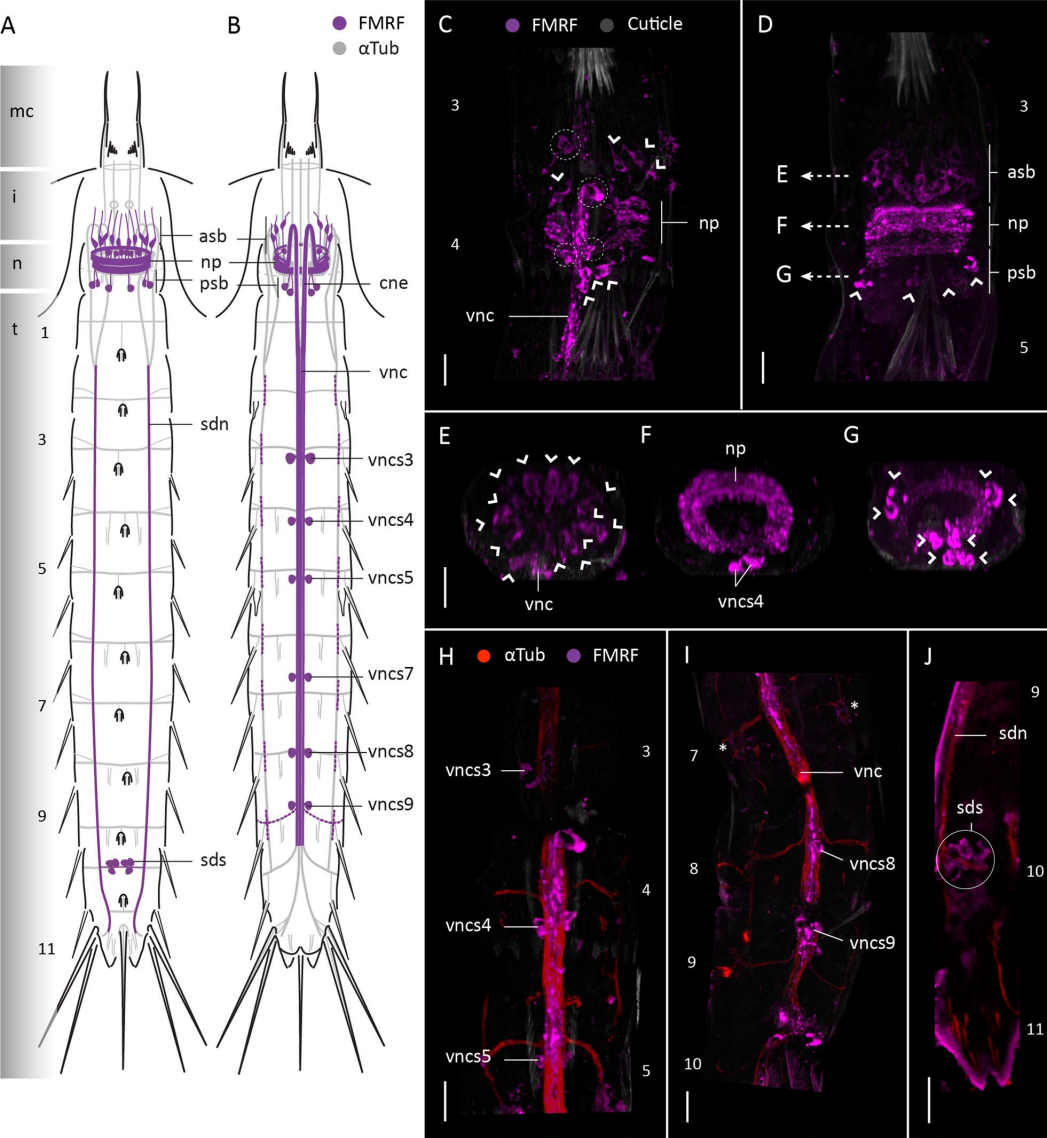
FMRF-LIR in the nervous system of *Franciscideres kalenesos.* Anterior is up in (**A–D, H–J**), dorsal is up in (**E–G**). **A**, **B**. Schematic representation, **A** dorsal view, **B** ventral view. FMRF-LIR is overlaid upon a background of α-tubulin-LIR; grey shading on the left margin marks the different body regions. Dotted lines mark weak FMRF-LIR. **B–G** Confocal Z-stack projections of specimens co-labelled with FMRFamide (**C–J**) and acetylated α-tubulin (**H–J**). Autofluorescence of the cuticle (grey) was kept as guidance in (**C–I**). Colour legend in (**C**) applies to (**D–G**), colour legend of (**H**) applies to (**I, J**). **C** detail of the brain neuropil, associated somata and ventral nerve cord, ventral view. Dashed circles mark the position of associated somata of the ventral nerve cord. **D** Same specimen as in (**C**), detail of the brain neuropil and associated somata, dorsal view. Arrows indicate the positions of the cross sections shown in (**E–G**). **E–G** Cross sections through the brain corresponding to the (**E–G**) levels indicated in (**D**). **H** Detail of the ventral nerve cord and associated somata of segments 3-5, ventral view. Note that the ventral nerve cord is broken in between segment 3 and 4. I, detail of the ventral nerve cord and associated somata in segments 7-10, ventral view; asterisks mark the areas where there is FMRF-LIR in the ventrolateral nerves. **J** Detail of the subdorsal nerves and the somata associated with the transverse neurite bundle of segment 10, dorsal view. Arrowheads mark the position of FMR-LIR somata of the brain. Scale bars: 10 μm. Abbreviations: asb, anterior somata of the brain; cne, convergent neurite; i, introvert; n, neck; np, neuropil; psb, posterior somata of the brain; sdn, subdorsal nerve; sds, subdorsal somata; t, trunk; vnc, ventral nerve cord; vncs, ventral nerve cord somata. Numbers refer to segments

FMRFamide-like-immunoreactivity (FMRF-LIR) was studied in four specimens of *F. kalenesos*. FMRF-LIR is consistently found in the brain neuropil, associated somata, ventral nerve cord and subdorsal nerves (Fig. 4). Weaker FMRF-LIR is present in the intersection areas between the ventrolateral nerves and transverse neurites of each segment, and in the transverse neurites of segment 9 (Fig. 4). Within the brain FMRF-LIR is localized in the neuropil (Fig. 4A–D, F). Multiple pairs of FMRF-LIR somata (ca. 10-12) similar in size are radially distributed in the anterior brain region (asb) (Fig. 4A–D, E). These neurons are interpreted as bipolar, with their somata seemingly projecting neurites into the anteriormost part of the neuropil as well as long neurites towards the introvert scalids. Additionally, five pairs of FMRF-LIR somata (two midventral, two midlateral and one subdorsal pair) are present in the posterior brain region connecting with the posteriormost part of the neuropil (Fig. 4A–D, G). The two convergent neurite bundles (cne) that emerge from the brain and the ventral nerve cord show several neurites with FMRF-LIR. The ventral nerve cord was labelled along its length until segment 10. Paired FMRF-LIR somata associated with the ganglia of the ventral nerve cord are present in segments 3-5, 7, 8 and 9 (Fig. 4A, B, H, I). All the somata are similar in size except for the somata of segment 3 which are larger (Fig. 4B, H). Moreover, at least three to four pairs of FMRF-LIR somata were found in subdorsal position on segment 10 associated with the transverse neurite bundle (Fig. 4 A, J).

### Neuroanatomy of *C. styx*

#### Acetylated α-tubulin-like immunoreactivity

**Fig. 5:**
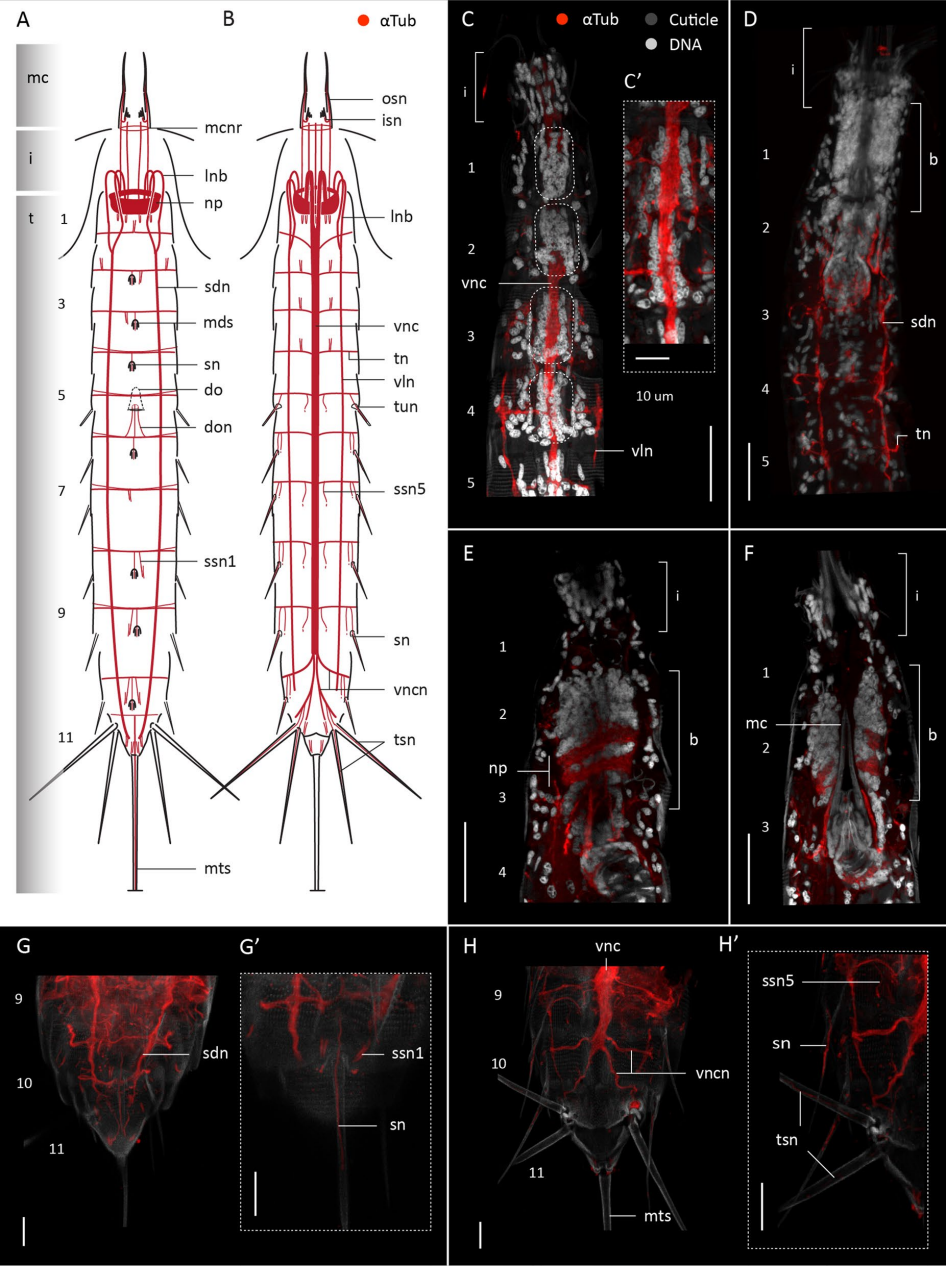
Acetylated α-tub-LIR in the nervous system of *Cateria styx.* Anterior is up in all panels. **A**, **B** Schematic representation in dorsal (**A**) and ventral (**B**) view. Grey shading on the left side marks the different body regions. For clarity, innervation of the introvert scalids have been omitted. Only the base of each of the middorsal spines is represented in (**A**). **C–H’** Confocal Z-stack projections of specimens co-labelled with acetylated α-tubulin (**C–H’**) and DAPI (**C–F**). Autofluorescence of the cuticle was kept for guidance. Colour legend in (**C**) applies to all panels. **C** Segments 1-5 ventral view, introvert everted. Dotted circles mark the position of the ventral nerve cord ganglia. **C’** Detail of the ventral nerve cord and ganglia of segments 3-4. **D** Same specimen as in (**C**) in dorsal view. **E** segments 1-4 showing a dorsal view of the brain, located in between segments 2-3, introvert everted. **F** Same specimen as in (**E**) with the focal point midway through the brain, showing the positon of the mouth cone. **G** Segments 9-11, dorsal view. **G’** Detail of the innervation of the middorsal spine and sensory spots type 1 of segment 10. **H** Segments 9-11, ventral view. **H’** Detail of the innervation of the ventrolateral spine, sensory spot type5 of segment 10 and terminal spines of segment 11, ventral view, right side. Scale bars: 30 μm (**C–F**); 10 μm (**G–H’**). Abbreviations: b, brain; do, dorsal organ; don, dorsal organ neurite; i, introvert; isn, inner oral styles neurite; lnb, longitudinal neurite bundle; mc, mouth cone; mcnr, mouth cone nerve ring; mds, middorsal spine; mts, midterminal spine; np, neuropil; osn, outer oral style neurite; sdn, subdorsal nerve; sn, spine neurite; ssn1/5, sensory spot type1/5 neurite; t, trunk; tn, transverse neurite bundle; tun, tube neurite; tsn, lateral terminal spine neurite; vln, ventrolateral nerve; vnc, ventral nerve cord; vncn, ventral nerve cord neurite bundle. Numbers refer to segments

Acetylated α-tub-LIR was studied in eighteen specimens of *C. styx.* In general, the α-tub-LIR pattern in *C. styx* agrees with the description provided for *F. kalenesos* and is therefore not included herein (Fig. 5), instead, only the diverging neuroanatomy is addressed.

The position of the brain in *C. styx* varies depending on the level of introvert retraction, reaching segment 5 when fully retracted. However, all the studied specimens with the introvert everted had the brain also located inside the trunk, in positions varying from segment 1-3 (Fig. 5D–F). The ventral nerve cord ganglia are paired, elongated and extend evenly along the segment length in parallel to the ventral nerve cord leaving short somata-free areas between segments (Fig. 5C–C’).

*C. styx* lacks a neck and therefore the cuticle of the anterior part of segment 1 continues directly into the soft cuticle of the introvert (Fig. 5A, B). This is different compared with *F. kalenesos*, which has a distinct neck region with a transverse neurite bundle encircling it.

Besides the lateral terminal and lateral terminal accessory spines, *C. styx* has a long midterminal spine (mts) in segment 11 that shows α-tub-LIR along its length. From the tip of the spine a long neurite extends towards the transverse neurite bundle of segment 11 (Fig. 5A).

*C. styx* has two types of sensory spots in the trunk (type 1 and type 5) with different α-tub-LIR patterns. Type 1 sensory spots show two neurites that connect to the transverse neurite bundle of the corresponding segment (ssn1); instead, sensory spots type 5 only have a single neurite (ssn5) (Fig. 5G–H’). Additionally, *C. styx* has a dorsal organ located in a middorsal position in between segments 5 and 6 that shows α-tub-LIR only in the proximal part (don) (Fig. 5A). Among all kinorhynchs, this organ is unique for *C. styx*. Details of the dorsal organ morphology are described and illustrated in Herranz et al. [9] and thus not included in this study.

#### Serotonin-like immunoreactivity

**Fig. 6:**
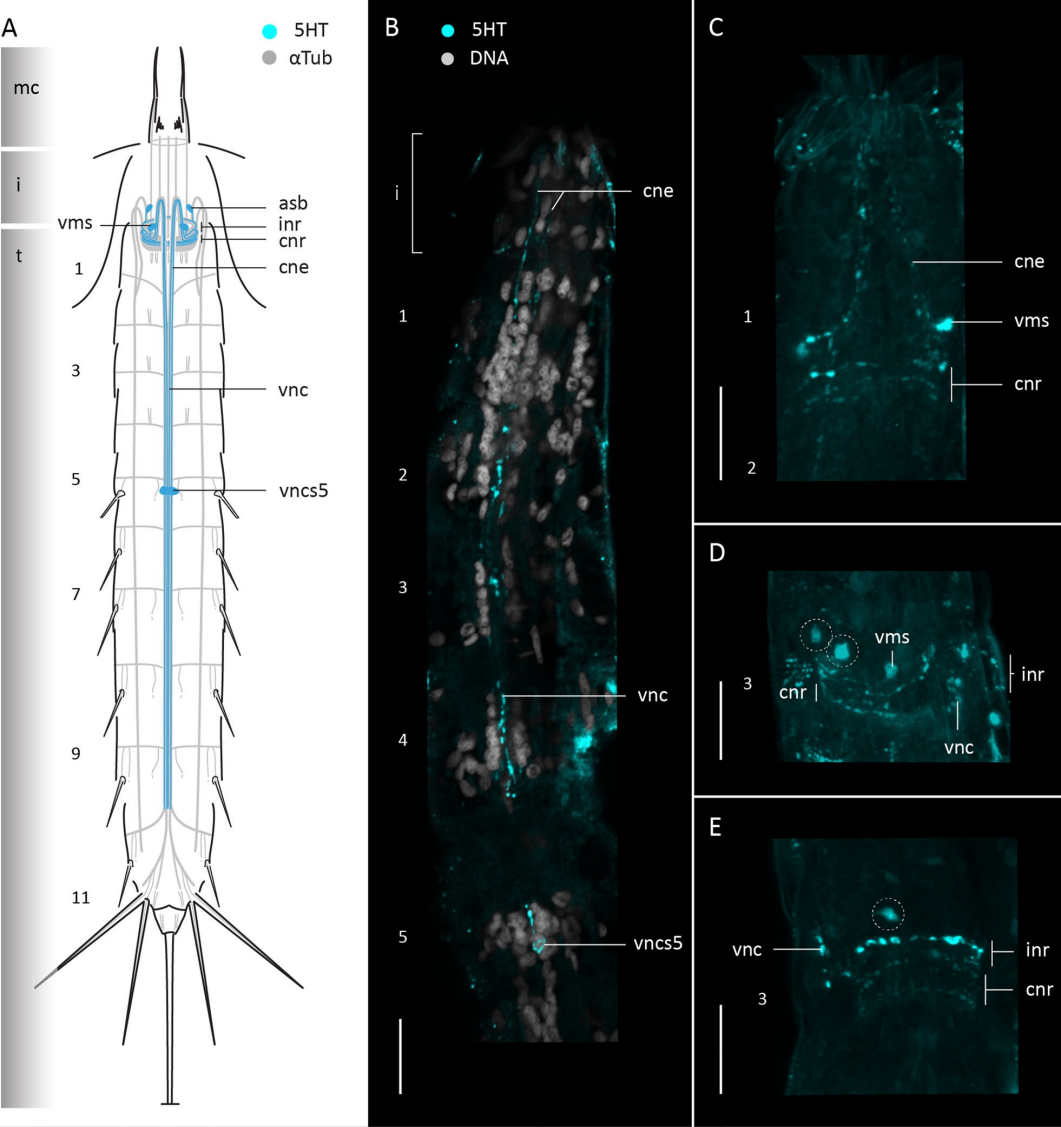
Serotonin-LIR in the nervous system of *Cateria styx*. Anterior is up in all panels. **A** Schematic representation, ventral view. Grey shading on the left side marks the different body regions. **B–E** Confocal Z-stack projections of specimens co-labelled with 5HT (**B–E**) and DAPI (**B**). Colour legend in (**B**) applies to all panels. **B** Detail of ventral nerve cord and associated somata, segments 1-5, ventral view, introvert everted. **C** Detail of brain neuropil, ventromedial somata and convergent neurites, ventral view, introvert everted. **D** Detail of brain neuropil and associated somata, lateral view. **E** Detail of brain neuropil and associated somata, laterodorsal view. Dashed circles mark the position of the 5HT-LIR somata associated to the brain in (**D–E**). Scale bars: 20 μm. Abbreviations: asb, anterior somata of the brain; cne, convergent neurite; cnr, 5HT-LIR complete nerve ring; i, introvert; inr, 5HT-LIR incomplete nerve ring; n, neck; t, trunk; vms, ventromedial somata of the brain; vnc, ventral nerve cord; vncs5, ventral nerve cord somata segment 5. Numbers refer to segments

Serotonin-like immunoreactivity was studied in twelve specimens of *C. styx*. All specimens show a common 5HT-LIR pattern only detected in the brain neuropil and the ventral nerve cord (Fig. 6A). In the brain neuropil there are ca. six immunoreactive neurites forming a ring, where the three first neurites are incomplete ventrally (Fig. 6A, C, D). The anteriormost incomplete ring is composed of neurites that extend from a pair of ventromedial somata (vms). The neurites of the two following posterior rings extend anteriorly towards the introvert and bend 180° to join the convergent neurite bundles (cne) that form the ventral nerve cord. Besides the ventromedial somata, there is an additional pair of 5HT-LIR somata within the anterior part of the brain located in laterodorsal position that projects neurites into the anterior part of the neuropil (Fig. 6A, C, D). No immunoreactive somata were found in the posterior brain region. Only one pair of 5HT-LIR somata was found associated with the ventral nerve cord (vncs) in segment 5 (Fig. 6A, B).

#### FMRFamide-like immunoreactivity

**Fig. 7:**
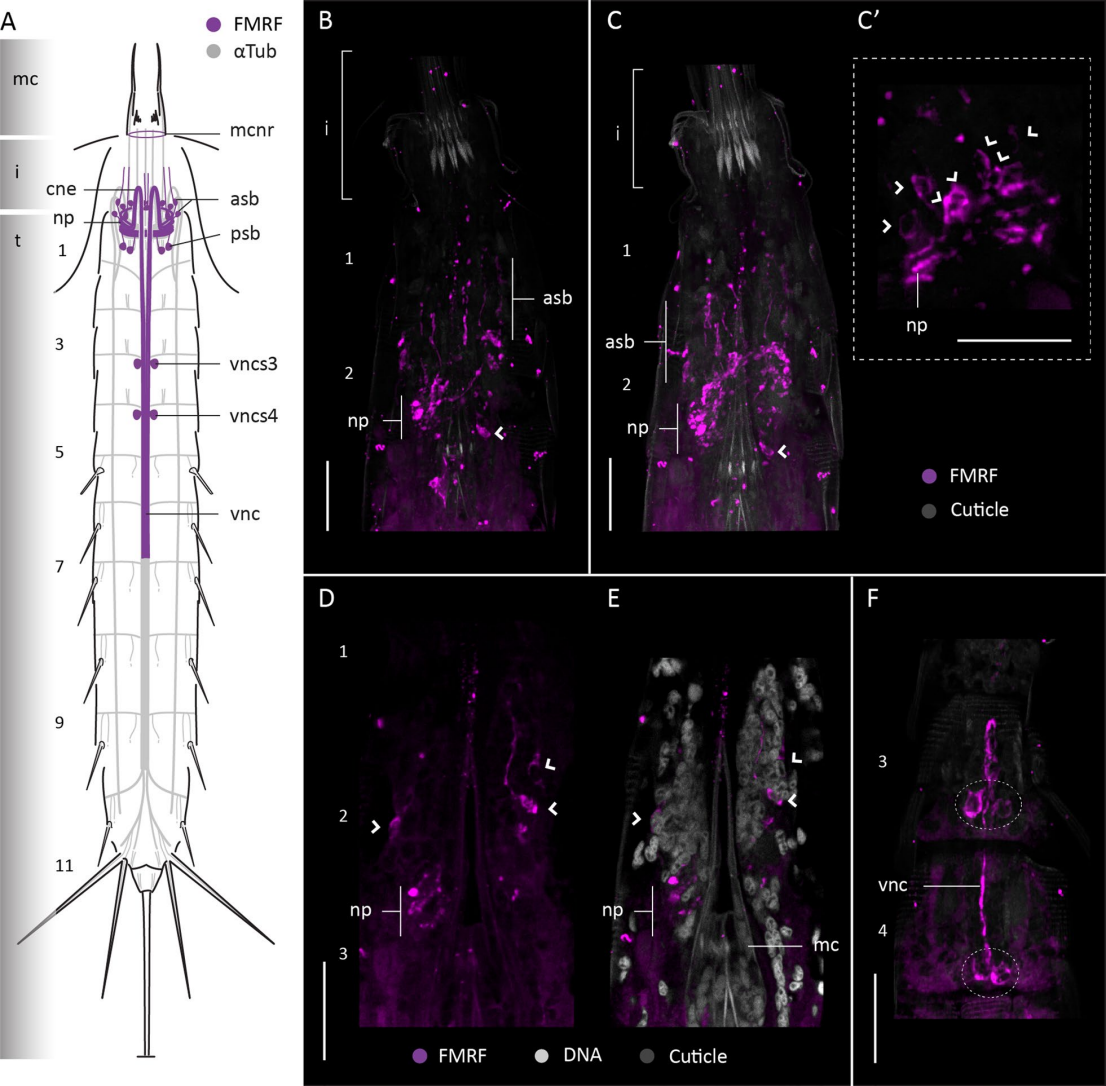
FMRF-LIR in the nervous system of *Cateria styx.* Anterior is up in all panels. **A** Schematic representation, ventral view. FMRF-LIR is overlaid upon a background representing α-tubulin-LIR; grey shading on the left margin marks the different body regions. **B–F** Confocal Z-stack projections of specimens co-labeled with FMRFamide (**B–F**) and DAPI (**E**). Autofluorescence of the cuticle (grey) was kept as guidance in (**B**, **C**, **E**, **F**). Colour legend in (**C**) applies to (**B**, **C**, **F**). **B** Detail of the brain neuropil and associated somata, ventral view. **C** Same specimen as in (**B**), detail of the brain neuropil and associated somata, dorsal view. **C’** Zoom into the brain neuropil in (**C**) showing the associated somata (marked by arrowheads), dorsal view. **D** Detail of the brain neuropil and associated somata in ventral view. **E** same image as in (**D**) including DAPI labelling. **F** detail of the ventral nerve cord and associated somata (dashed circles) of segments 3-4, ventral view. Arrowheads mark the position of FMR-LIR somata of the brain in (**C’–E**). Scale bars: 20 μm. Abbreviations: asb, anterior somata of the brain; cne, convergent neurite; i, introvert; n, neck; np, neuropil; psb, posterior somata of the brain; t, trunk; vnc, ventral nerve cord; vncs3/4, ventral nerve cord somata segment 3/4. Numbers refer to segments

FMRF-LIR was studied in six specimens of *C. styx.* Stainings of the posteriormost trunk segments [7–11] were not successful. FMRF-LIR is consistent in the brain neuropil, associated somata, base of the mouth cone and the ventral nerve cord (Fig. 7). The brain neuropil shows multiple FMRF-LIR neurites along its full width (Fig. 7A–C). Associated with the neuropil, multiple sets of somata are located in the anterior and posterior brain regions but fewer than in *F. kalenesos*. The anterior brain region shows at least six pairs of bipolar somata (asb), radially arranged and similar in size, that project neurites into the anterior part of the neuropil as well as anteriorly towards the introvert (Fig. 7A–E). The posterior brain region only shows two pairs of ventromedial somata (psb) which connect with the posterior part of the neuropil (Fig. 7A–C). FMRF-LIR was also detected as a ring at the base of the mouth cone, within the mouth cone nerve ring (mcnr). The ventral nerve cord shows FMRF-LIR at least from segments 1-7. FMRF-LIR pairs of somata are only situated at the posteriormost region of segments 3-4 (Fig. 7A, F), and seem to be part of the ventral nerve cord ganglia.

### Neuroanatomy of *Z. yong*

#### Acetylated α-tubulin-like immunoreactivity

**Fig. 8:**
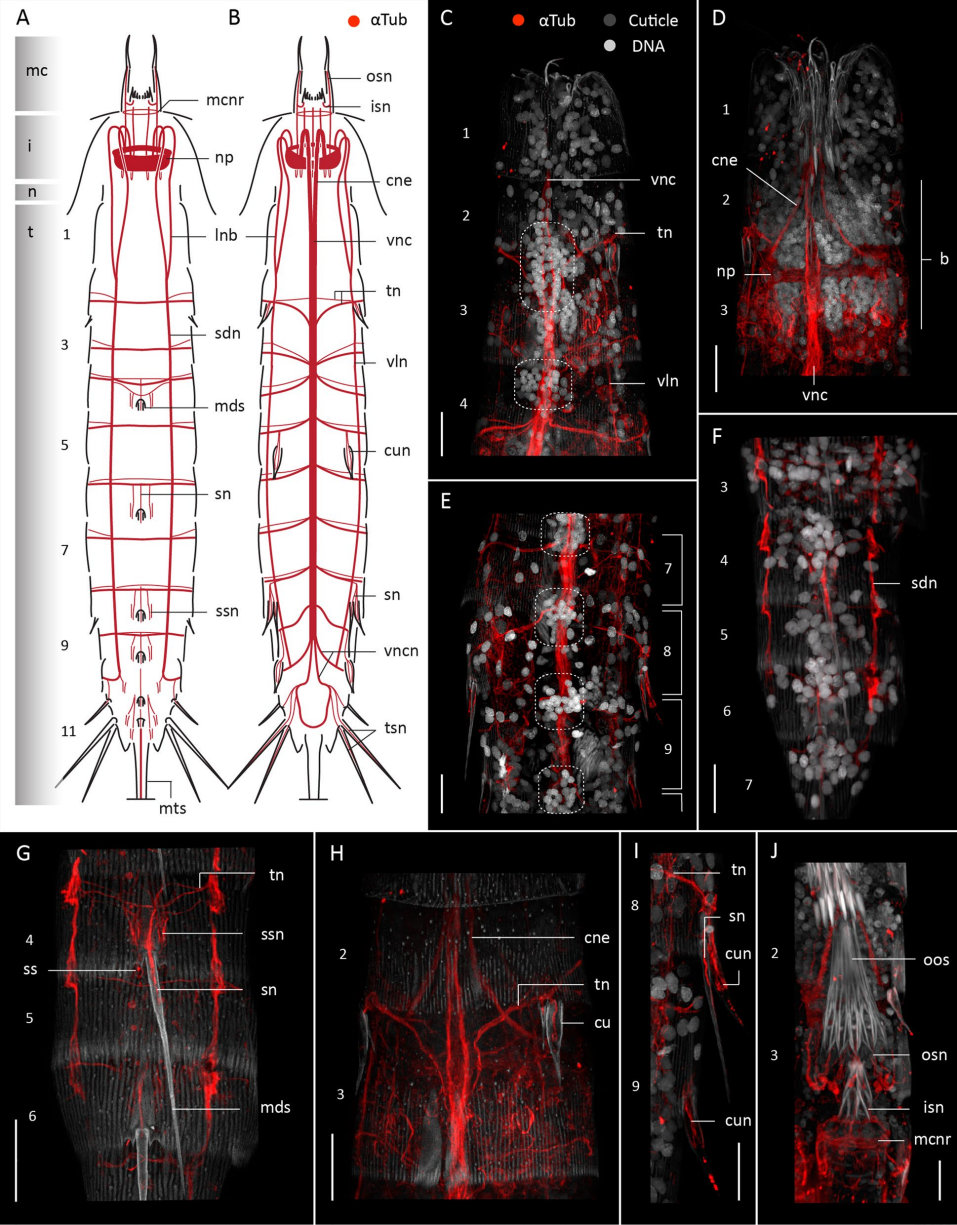
Acetylated α-tub-LIR in the nervous system of *Zelinkaderes yong*. Anterior is up in all panels. **A**, **B** Schematic representation in dorsal (**A**) and ventral (**B**) view. Grey shading on the left side marks the different body regions. For clarity, innervation of the introvert scalids have been omitted. Only the base of each of the middorsal spines is represented in (**A**). **C–J** Confocal Z-stack projections of specimens co-labelled with acetylated α-tubulin (**C–J**) and DAPI (**C–F**, **I**, **J**). Autofluorescence of the cuticle was kept for guidance. Colour legend in (**C**) applies to all panels. **C** Segments 1-4 ventral view, introvert retracted. Dotted areas mark the position of the ventral nerve cord ganglia. Note the length of the ganglia between segment 2-3 and absence of ganglia in segment (1) **D** Detail of the brain, convergent neurites and ventral nerve cord, segments 1-3, introvert retracted. **E** Segments 7-9 showing the ventral nerve cord, ganglia and transverse neurites. Brackets mark the limits of each segment. Note the position of some ganglia and origin of transverse neurites is intersegmental. **F** Segments 3-6, dorsal view. **G** Detail of the innervation of the middorsal spines and sensory spots of segments 4-6, dorsal view. **H**.Same specimen as in (**C**) detail of segments 2-3, ventral view, note the position of the transverse neurite of segment (2) **I** Detail of the innervation of cuspidate and acicular spines of segments 8-9, left side, ventral view. **J** Detail of the mouth cone innervation. Scale bars: 20 μm. Abbreviations: b, brain; cne, convergent neurite; cu, cuspidate spine; cun, cuspidate spine neurite; i, introvert; isn, inner oral style neurite; lnb, longitudinal neurite bundle; mc, mouth cone; mcnr, mouth cone nerve ring; mds, middorsal spine; mts, midterminal spine; np, neuropil; osn, outer oral style neurite; sdn, subdorsal nerve; sn, spine neurite; ssn, sensory spot neurite; t, trunk; tn, transverse neurite bundle; tsn, lateral terminal spine neurite; vln, ventrolateral nerve; vnc, ventral nerve cord; vncn, ventral nerve cord neurite bundle. Numbers refer to segments

Acetylated α-tub-LIR was studied in twelve specimens of *Z. yong*, all of them showing identical patterns (Fig. 8). The pattern of α-tub-LIR in *Z. yong* is very similar to *F. kalenesos* and *C. styx*, and therefore this description will only focus on the differential neuroanatomy.

The position of the brain in *Z. yong* varies depending on the degree of eversion/retraction of the introvert. In *F. kalenesos* and *C. styx* the brain can remain inside the anterior trunk segments or the neck even when the introvert is everted. All specimens of *Z. yong* had the introvert withdrawn (Fig. 8D) and thus the position of the brain relative to the everted introvert could not be examined (Fig. 8A, B shows introvert and mouth cone extended to ease comparisons among species, the position of the brain herein should not be considered).

The ventral nerve cord in *Z. yong* extends from segment 1-10. Clusters of nuclei are distinct in most segments forming paired ganglia (dashed areas in Fig. 8C, E), except in segments 1 and 10 (Fig. 8C). Unlike *F. kalenesos* and *C. styx*, most of the ventral nerve cord ganglia are located in the anteriormost part of each segment in *Z. yong* and in some segments the ganglia seem to be placed in the intersegmental region (Fig. 8C, E). Except for the very elongated ganglia situated between segments 2 and 3, the ganglia of *Z. yong* are conspicuously round and compact. The elongated ganglia between segments 2 and 3 are almost as long as a segment, with nuclei scattered along the inter-ganglionic region and placed so close to each other that seem to form a single ganglion (Fig. 8C). In addition, less distinct aggregations of somata can be found in a middorsal position in each segment, especially in those segments with a middorsal spine (Fig. 8F).

Additional differences from *F. kalenesos* and *C. styx* include the transverse neurite bundles in the trunk (tn) of *Z. yong* which are only present in segments 2-9. They emerge from left and right sides of the ventral nerve cord at the level of the ganglia and encircle each segment. The transverse neurite bundles from segment 2 emerge from the ventral nerve cord in the anteriormost part of segment 3 and extend diagonally towards the posteriormost part of segment 2 (Fig. 8B, C, H). In segment 3 the transverse neurites are instead located in the posteriormost part of the segment. In segments 4-9 the transverse neurites are situated in the anteriormost part of each segment (Fig. 8B, E). The ventral nerve cord bifurcates in segment 10 into four neurite bundles (vncn) (Fig. 8B). Two of these neurite bundles extend laterally and encircle segment 10 while the other two extend towards segment 11 and form a ventral loop (Fig. 8B).

As in *F. kalenesos* and *C. styx*, *Z. yong* shows intrinsic α-tub-LIR in all the trunk spines. In addition, *Z. yong* has cuspidate spines (cu), which also show α -tub-LIR. Each cuspidate spine shows several neurites (cun) that extend radially from the point where the spine starts narrowing down towards the base of the spine and connect to the transverse neurite of the corresponding segment (Fig. 8, H, I). As in *C. styx*, *Z. yong* has a midterminal spine that shows α-tub-LIR (Fig. 8A).

#### Serotonin-like immunoreactivity

**Fig. 9:**
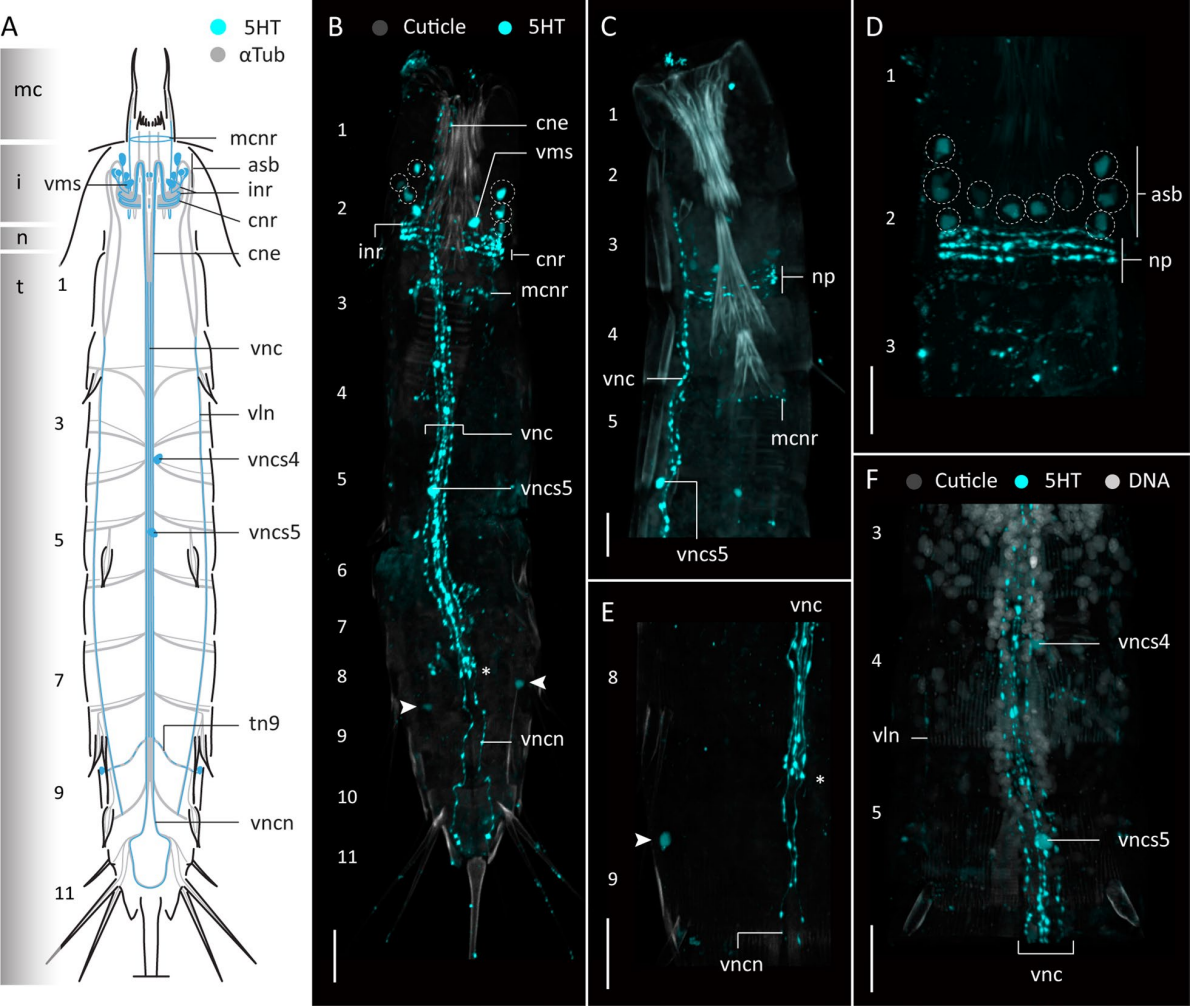
Serotonin-LIR in the nervous system of *Zelinkaderes yong*. Anterior is up in all panels. **A** Schematic representation, ventral view. Grey shading on the left side marks the different body regions. **B–F** Confocal Z-stack projections of specimens co-labeled with 5HT (**B–F**), and DAPI (**F**). Autofluorescence of the cuticle was kept as guidance. Colour legend in (**B**) applies to (**C–E**). **B** 5HT-LIR overview, ventral view. **C** Segments 1-5, lateral view, left side, introvert withdrawn. **D** Detail of brain neuropil and associated somata, dorsal view. **E** Segments 8-9, detail of the ventral nerve cord 5HT-LIR neurites and lateroventral 5HT-LIR somata, ventral view. **F** Segments 3-5, detail of ventral nerve cord and associated 5HT-LIR somata, ventral view. Arrows mark the position of the immunoreactive somata of segment 9 in (**B**, **E**). Dashed circles mark the position of the 5HT-LIR somata associated to the brain in (**B–D**). Scale bars: 20 μm. Abbreviations: asb, anterior somata of the brain; cne, convergent neurite; cnr, 5HT-LIR complete nerve ring; i, introvert; inr, 5HT-LIR incomplete nerve ring; mcnr, mouth cone nerve ring; n, neck; np, neuropil; t, trunk; tn, transverse neurite; vln, ventrolateral nerve; vms, ventromedial somata of the brain; vnc, ventral nerve cord; vncs, ventral nerve cord somata; vncn, ventral nerve cord neurite. Numbers refer to segments

Serotonin-like immunoreactivity was studied in six specimens of *Z. yong*. 5HT-LIR is present in the brain neuropil, associated somata, ventral nerve cord and ventrolateral nerves (Fig. 9). Within the neuropil there are at least five immunoreactive rings where only two are incomplete ventrally (inr) (Fig. 9A). The first ring extends from a pair of 5HT-immunoreactive somata in ventromedial position (vms) (Fig. 9A, B). Five additional 5HT-LIR somata (asb) are present in the anterior brain region and project neurites into the neuropil (Fig. 9A, B, D). The ventral nerve cord shows at least five 5HT-LIR neurites that extend from segments 1-8, from which only two neurites extend posteriorly towards segment 11, forming a loop (Fig. 9A, B, E). Only a couple of 5HT-LIR somata were found associated with the ventral nerve cord (vncs), one on segment 5 and another one on segment 4, although not consistently present in all the studied specimens (Fig. 9A, B, E). Additionally, a pair of 5HT-LIR somata is present in a lateroventral position in segment 9 (Fig. 9A, B, E). Weak 5HT-LIR correlated with the transverse neurites of segment 9 was detected.

#### FMRFamide-like immunoreactivity

**Fig. 10:**
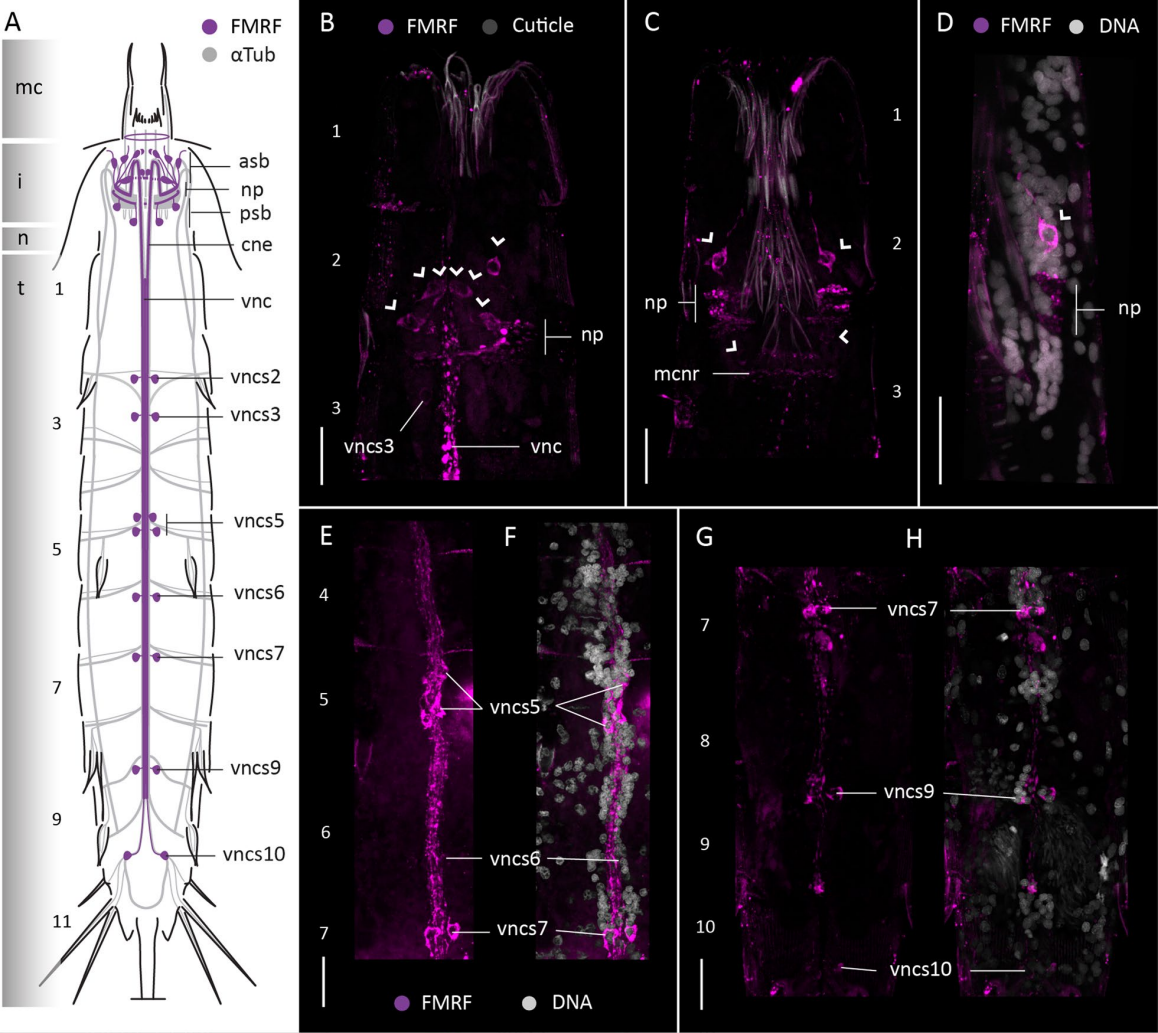
FMRF-LIR in the nervous system of *Zelinkaderes yong.* Anterior is up in all panels. **A** Schematic representation, ventral view. FMRF-LIR is overlaid upon a background representing α-tubulin-LIR. Grey shading on the left margin marks the different body regions. **B–H** Confocal Z-stack projections of specimens co-labelled with FMRFamide (**B–H**) and DAPI (**D**, **F**, **H**). Colour legend in (**B**) applies to (**C**), legend in (**E**, **F**) applies to (**G**, **H**). **B** Detail of the brain neuropil, associated somata and ventral nerve cord, ventral view. **C** Same specimen as in (**B**) with a deeper focal point showing the brain neuropil and the bipolar somata, dorsal view. **D** Zoom into the brain neuropil in (**C**) showing a bipolar somata. **E**, **F** Ventral nerve cord and associated somata of segments 4-7, ventral view. **G**, **H** Ventral nerve cord and associated somata of segments 7-10, ventral view. Arrowheads mark the position of FMR-LIR somata of the brain in (**B–D**). Scale bars: 20 μm. Abbreviations: asb, anterior somata of the brain; cne, convergent neurite; i, introvert; n, neck; np, neuropil; psb, posterior somata of the brain; t, trunk; vnc, ventral nerve cord; vncs, ventral nerve cord somata. Numbers refer to segments

FMRF-LIR was studied in five specimens of *Z. yong* showing a consistent pattern in the brain neuropil and ventral nerve cord (Fig. 10A). Six pairs of FMRF-LIR somata (asb) are connected with the brain neuropil in the anterior region, and two pairs of somata (psb) in the posterior brain region (Fig. 10A–D). Some of the anterior somata are bipolar neurons with one neurite extended towards the introvert and another towards the neuropil (Fig. 10C, D). FMRF-LIR was also detected as a ring at the base of the mouth cone constituting a part of the mouth cone nerve ring (mcnr). The ventral nerve cord shows FMRF-LIR along its length, paired associated FMRF-LIR somata are present in segments 2, 3, 5 (two pairs), 6, 7, 9 and 10 (Fig. 10A, E–H). The position of the FMRF-LIR somata of the ventral nerve cord seem to be correlated with the position of the ganglia, except in segment 10 (Fig. 10A, F, H).

## Discussion

### Conserved neuroarchitecture across Kinorhyncha

Comprehensive neuroarchitecture data based on CLSM reconstructions of α-tubulin immunoreactivity is now available for three cyclorhagid (*Cateria, Echinoderes, Zelinkaderes*) and four allomalorhagid genera (*Dracoderes*, *Franciscideres*, *Pycnophyes*, *Setaphyes*) [9, 10, 30, this study]. From these studies we can confirm that the overall neuroarchitecture is conserved across Kinorhyncha showing: (1) circumpharyngeal brain with somata-neuropil-somata organization; (2) ten longitudinal neurite bundles arising from the brain and converging in the first trunk segments into two subdorsal, two ventrolateral and one midventral nerve cord, all extending along the trunk to reach at least segment 8; (3) ventral nerve cord with segmentally arranged ganglia in at least segments 2-8; (4) at least one transverse neurite bundle emerging laterally from the ventral nerve cord and encircling each segment; (5) introvert scalids and mouth cone styles showing neurites that connect with the anterior and posterior parts of the neuropil, respectively; (6) sensory neurons of acicular spines, cuspidate spines and sensory spots (numbers and positions may vary) connecting with the transverse neurite bundle of the corresponding segment.

### Serotonin and FMRFamide-like immunoreactivity across Kinorhyncha

Immunohistochemical studies tracing serotonin-LIR (5HT-LIR) and FMRF-LIR in all kinorhynch genera examined so far showed some conserved patterns [9, 10, 26, 30, this study]. 5HT-LIR is always detected in the mouth cone and brain, showing at least two incomplete and a minimum of two complete rings in the neuropil. There are always 5HT-LIR somata in the anterior brain region, but the number varies across genera. Cyclorhagids tend to show more than three pairs of 5HT-LIR somata while allomalorhagids generally show three or less pairs [9, 10, 26, 30, this study]. However, the ventromedial pair of somata seems to be a common trait across Kinorhyncha. 5HT-LIR somata in the posterior brain region have only been detected in the allomalorhagid species *Franciscideres kalenesos*, *Pycnophyes ilyocryptus* (Higgins, 1961) and *Setaphyes kielensis* (Zelinka, 1928) [10, 30, this study]. The ventral nerve cord shows 5HT-LIR along its length in all kinorhynchs, but in the more posterior trunk segments it either forms a loop as in *Antygomonas* and *Zelinkaderes* [26], this study) or a bifurcation as in the remaining studied genera. Initially, it was assumed that the 5HT-LIR loop was associated with the presence of a midterminal spine, however, such a loop is lacking in *C. styx* even though it has a midterminal spine. Interestingly, the midterminal spine in *C. styx* also differs from those in *Antygomonas* and *Zelinkaderes* species by not showing any associated musculature and almost resembling a middorsal spine (as seen in segment 11 in *F. kalenesos*). This suggests an independent origin of the midterminal spines in *Cateria* and other kentrorhagids, and supports that *Cateria* should not be considered a part of the Kentrorhagata [27]. The number and position of 5HT-LIR somata associated with the ventral nerve cord vary among genera. Only the generally unpaired and conspicuous somata situated midventrally in segment 5 are found in all examined kinorhynchs.

FMRFamide-like peptides comprise a large and diverse family of neuropeptides with multiple functions, hence, direct comparison of FMRF-LIR across species is problematic due to the variety of potentially labelled neuropeptides. Overall, the FMRFamide-like immunoreactive pattern seems to be consistent in the ventral nerve cord and in the brain neuropil in all kinorhynchs studied so far. Multiple FMRF-LIR somata are associated with the anterior and posterior brain regions, however the number is quite variable among genera [9, 10, this study]. Most of these somata are associated with the anterior brain region, from which several pairs are bipolar neurons, each extending a neurite towards the introvert [9, 10, this study]. The ventral nerve cord usually shows FMRF-LIR paired somata in most segments, however their distribution is quite variable.

### Divergent neural characters in aberrant kinorhynchs

The kinorhynch brain is usually positioned inside, and attached to, the anteriormost part of the introvert. Thus, if the introvert is retracted the brain is dragged into the trunk [6, 7, 9, 39]. In *C. styx* and *F. kalenesos* the position of the brain is not fully synchronised with the movement of the introvert, and therefore, when the introvert is everted, the brain can stay inside the neck or segments 1-2 in *F. kalenesos* and in segments 2-3 in *C. styx.* This could not be confirmed for *Z. yong* since all the studied specimens had the introvert retracted. Ultrastructural studies of *Echinoderes* described the brain as being attached to the introvert epidermis between the first scalid ring and the basis of the mouth cone [6, 39]. One of the characteristic modifications in aberrant kinorhynchs is the elongation of the primary spinoscalids, resembling tentacle-like appendages, and also, the elongation of the cuticular area joining the introvert and the mouth cone [27]. Assuming that the brain in *C. styx* and *F. kalenesos* is also attached in the area between the primary spinoscalids and the mouth cone base, if this area is much longer than in other non-aberrant kinorhynchs (e.g., *Echinoderes*, *Dracoderes*), it would mean that the brain could physically be placed further from the introvert spinoscalids. This would explain the less correlated movement of the brain in respect to the introvert, as observed in *C. styx* and *F. kalenesos*. Additionally, the length of the neurites connecting the spinoscalids and oral styles with the brain neuropil are very long and convoluted, showing a slack when the introvert is fully retracted in the trunk. This allows a more flexible position of the brain along the anterior-posterior axis (Fig. 2C, D).

The ventral nerve cord of *C. styx*, *F. kalenesos* and *Z. yong* exhibits one additional pair of ganglia in segment 9 before branching out in the last two segments. A similar, organization of the ventral nerve cord is found in *Tubulideres* and *Antygomonas* (Herranz unpublished data). The ventral nerve cord of *Z. yong* lacks distinct ganglia and transverse neurite bundles in segment 1; however, the large posterior ganglia in segment 2 could be considered homologous to the large ganglia of segment 1 present in all the other studied kinorhynch genera. In this case, the ganglia in *Z. yong* are posteriorly displaced one to one and a half segment length. In addition, the transverse neurite bundles do not correlate with the position of the ganglia in segments 2-5 (Fig. 8C). This particular arrangement of the ventral nerve cord ganglia and transverse neurite bundles is shared with other *Zelinkaderes* species (Herranz unpublished data) and may be an autapomorphy of the genus.

There is also some variation in the level of aggregation of the ventral nerve cord somata into distinct ganglia. Sometimes, the ganglia are so elongated that they occupy most of the segment length reducing the somata-free connective area to a minimum. This almost gives the appearance of a medullary cord, as seen in *C. styx* but also described in some non-aberrant species (e.g., *Echinoderes* [39], *Dracoderes*, *Antygomonas* and *Tubulideres* [10, 26], Herranz unpublished data).

Two pairs of ring-like structures associated with the brain show α-tub-LIR in *F. kalenesos* (Fig. 2C, D). These structures are conspicuous, consistent in all the studied specimens, and have never been detected in any other kinorhynch species studied so far. Based on the shape, size and intensity of the labelling, each ring is here interpreted to be a potential coiled cilium or a bundle of cilia surrounding a single cell. Ciliated cells without a connection to the exterior have previously been described ultrastructurally in several Pycnophyidae species as part of cephalic unpigmented photoreceptive organs [6, 47]. These organs are paired, located at the base of the primary spinoscalids and are composed of an enveloping cell and a ciliary receptor cell [47]. Even though, the number or position of the ring-like structures seen in *F. kalenesos* do not exactly correspond with these organs, it seems likely that they could also have a photoreceptory function. However, ultrastructural studies would be necessary to confirm if these rings are part of a ciliary sensory organ. Ring-like structures (described as donut-like) with highly similar α-tub-LIR appearance were detected in the brain of protodrilid annelids [48]. These donut-like structures were described with TEM as part of ciliary photoreceptor organs composed of an enveloping cell and a multiciliated receptor cell, with few or multiple cilia coiled to form a bundle [48, 49]. The immunoreactive signal shown in protodrilids is very similar to the signal found in *F. kalenesos* suggesting that the ring-structure in the latter might also be ciliary.

### Segmental mismatch

**Fig. 11:**
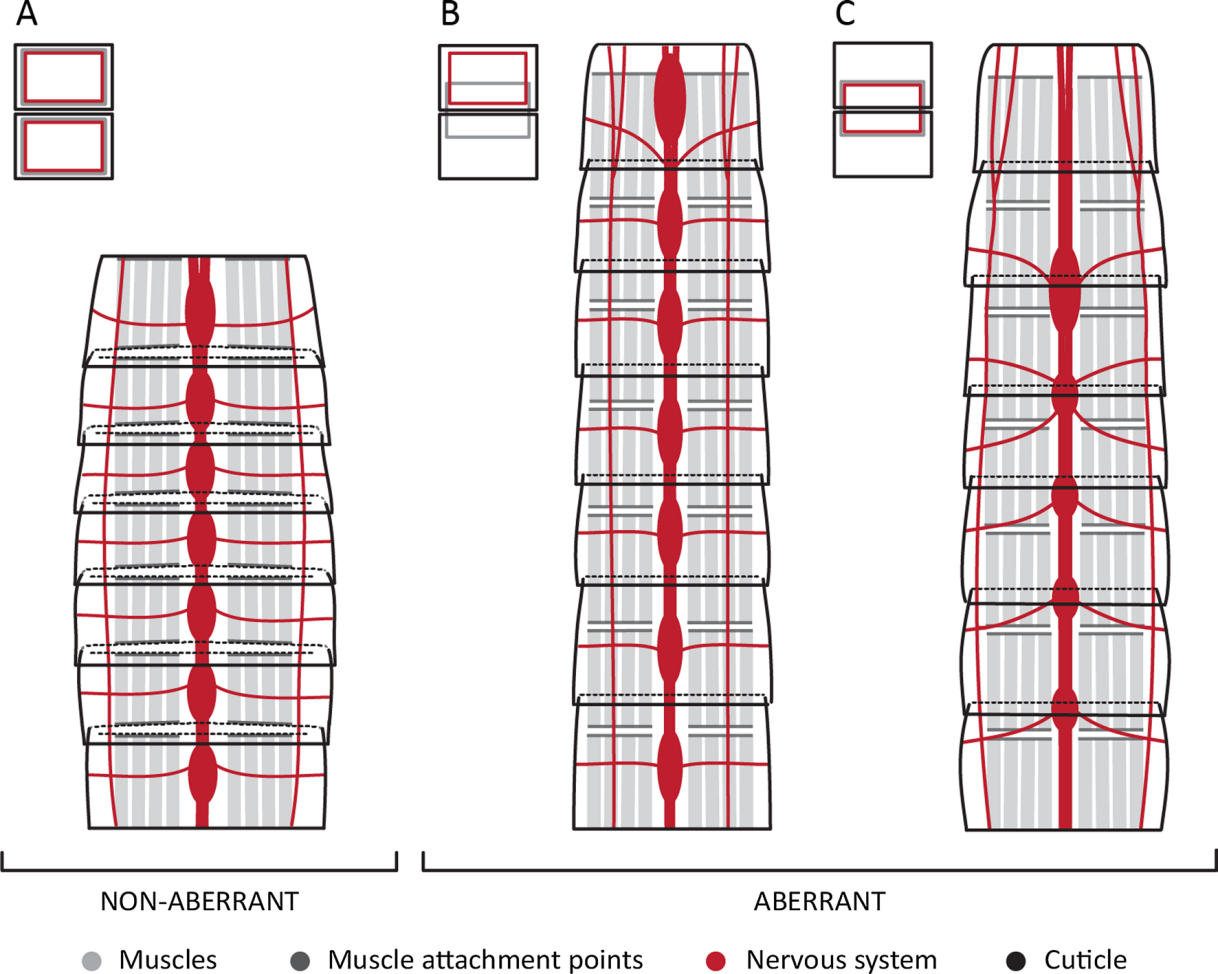
Comparison of musculature and nervous system arrangement between aberrant and non-aberrant kinorhynchs in respect to the external trunk segmentation. All illustrations represent the anterior seven trunk segments in ventral view. Icons on the top left corner of (**A–C**) represent segmental match or mismatch of musculature and nervous system elements in respect to the external segments (see legend for colours). **A** Aligned musculature and nervous system in respect to the external segmentation in non-aberrant kinorhynchs. **B** Aligned nervous system and misaligned musculature in respect to the external segmentation in *C. styx* and *F. kalenesos.* **C** Misaligned musculature and nervous system in respect to the external segmentation in *Zelinkaderes* species

All kinorhynchs, including the aberrant groups, show segmental patterning of the tegumental plates, nervous system and musculature (Fig. 11A). However, in aberrant kinorhynchs these traits are not fully correlated (matched), sometimes showing a posterior shift of the segmental longitudinal muscles of the trunk relative to the tegumental plates [27] (Fig. 11B, C). This positional shift has been associated with the disappearance of the anterior cuticular thickenings of the segments, named pachycycli, where the longitudinal muscles of the trunk usually attach [27]. In the nervous system, we find a posterior shift in the position of the ventral nerve cord ganglia and transverse neurites in *Z. yong* (following the musculature) (Fig. 11C), whereas such a shift was not found in *Cateria* and *Franciscideres* (Fig. 11B).

Segmental mismatch between elements of different organ systems in the same organism has also been reported in tardigrades, arthropods, annelids and chordates (summarized by [50, 51]). In arthropods, examples of discordant segmental structures have been described in extant and extinct lineages. Within extant species it has been described in crustaceans such as the tadpole shrimp [52, 53]. In addition, it is particularly frequent in the trunk of myriapods where dorsal and ventral structures such as dorsal sclerites, nerve ganglia and leg pairs seem to be misaligned [54, 55], reviewed in [52]. In extinct species it has been reported from the arthropod stem-groups Fuxianhuiida and Trilobita [56, 57]. These mismatches seem to have occurred multiple times through arthropod evolution [52]. Additionally, some arthropods (e.g., crustaceans, insects and spiders) show at the cellular level segmental units, named parasegments, with the same length of a segment but shifted half a segment with respect to the external boundaries [51]. However, these units are usually transient, formed by segment polarity genes and present during development [2, 51].

Segmental mismatch in Kinorhyncha may also have evolved several times. Aberrant kinorhynchs show different types of mismatches either involving one organ system as in *Cateria* and *Franciscideres* or several as in *Z. yong* (Fig. 11). These differences suggest divergent evolutionary trajectories of aberrant kinorhynchs. This hypothesis is supported by the phylogenetic study by Sørensen et al. [40] where the aberrant kinorhynchs are highly derived and distantly related within Kinorhyncha. This would imply that the segmental mismatch has happened at least twice independently during kinorhynch evolution and thus a “matched” segmentation would represent the ancestral condition. Interestingly, all the aberrant genera are mainly found in sandy substrates rather than mud, supporting that the worm-like body plan might represent an adaptation to the interstitial environment [27, this study].

### Developmental considerations

When seeking to understand the evolution of a segmented body plan developmental biologists not only put emphasis on spatial coordination but also in the temporal coordination of segment formation [3]. Kinorhynchs are direct developers, and their embryonic development is very poorly studied, and restricted to a couple of light microscopical studies by Kozloff [11, 12] on a single species. However, post-embryonic developmental observations in multiple genera demonstrate that the first juvenile stage (J1) shows a clear external segmentation and will go through five additional juvenile stages prior to becoming adult [6, 7, 11–13, 44, 58–61]. The first juvenile stage hatches from the egg with a head, a neck, and eight or nine seemingly differentiated trunk segments of which the last segment is the anlagen of the two to three remaining segments (summarized by Neuhaus, [7]). In any given kinorhynch the new segments generate from a subcaudal differentiation zone by elongation of the terminal segment and posterior transverse division [7]. Yet, the cellular and anatomical differentiation during this post-hatching development is unknown as is the developmental process before hatching. Interestingly, in kinorhynchs there might be some time-space independence in the post-hatching development of the longitudinal *versus* dorsoventral muscle sets in the trunk [29]. Myoanatomical studies of *Setaphyes kielensis* (*Pycnophyes kielensis* in [29]) showed that in an eight-segmented juvenile specimen (J1) all 11 groups of dorsoventral muscles were already present, whereas the longitudinal muscles of the three posterior segments may generate post-hatching [29].

So far, the nervous system in all the studied kinorhynchs follow a segmental pattern with a ganglionated ventral nerve cord present in segments 1-8, 2-8 or 1-9 depending on the genus [9, 10, 30, this study]. However, from segments 8 or 9, the ventral nerve cord branches into 4-6 neurite bundles that innervate the posteriormost segments [9, 10, this study]. No nervous system studies have examined pre- or post-hatching developmental stages so far, but we speculate that the seriality of the ganglionated ventral nerve cord is already established pre-hatching and is correlated with the observed seriality in teguments and dorsoventral muscles. Our study corroborates this by finding nine pairs of ganglia (rather than eight) in the adult stage of *C. styx*, *Z. yong* (this study), and *Z. brightae* (Herranz unpublished data), matching the fact that these genera have also been found to hatch with nine segments (instead of eight) [7, 44, 59, 61]. Interestingly, the posteriormost 2-3 segments that develop post-hatching, never establish a similar ganglionated ventral nerve cord despite developing segmental musculature [10]. This indicates a different segmentation process pre- and post-hatching.

Similarly, arthropods, annelids and chordates can generate segments in a different way between body regions, and with different temporal sequences [51, 52, 62]. Particularly, in arthropods with anamorphic development, segment differentiation has been reported to continue in different form and degree post-embryonically (summarized in [63]).

Whether the appearance of the segmental organ systems in Kinorhyncha comes from undifferentiated segmental precursors, or if this process is synchronized during embryogenesis remains elusive. In order to fill the gap of knowledge, future research should be focused on ontogenetic studies looking for temporal coordination of segmentation during development, cell lineage studies, and gene expression studies focused on segmentation-associated genes.

## Conclusions

Our study demonstrates that despite their aberrant appearance, worm-like kinorhynchs show a clearly segmentally arranged nervous system, with a ganglionated ventral nerve cord, and transverse neurite bundles encircling each segment. This pattern is congruent with the conserved nervous system architecture shown in all other studied kinorhynchs, indicating this to be the ancestral condition for Kinorhyncha. However, some variations deviating from the typical neuroanatomy (excluding those associated with sensory organ number and position) could be found in one of the aberrant kinorhynchs. *Z. yong* shows a posterior shift of the ganglia and the transverse neurites that encircle each segment, not correlating with the external segmentation of the trunk but correlating with the posteriorly shifted musculature. This result emphasizes the morphological differences between *Z. yong* and both *Franciscideres* and *Cateria*, and supports that the worm-like appearance of *Zelinkaderes* might have evolved independently from the aberrant shapes of *Franciscideres* and *Cateria*. The spatial correlation of tegumental plates and nervous system present in all non-aberrant kinorhynchs as well as in *Cateria* and *Franciscideres* suggests that this segmental correlation was present in the kinorhynch ancestor.

## Methods

### Sampling

Specimens of *Cateria styx* were collected intertidally at Cavaleiro beach in Macaé, Brazil (22^o ^21′ 59″ S, 41^o ^46′ 27″ W) in March 2015. Sediment samples were collected from the bottom of ca. 50 cm deep holes dug on the beach located ca. 3 m below the high tide mark. Specimens of *Franciscideres kalenesos* were collected subtidally by hand from the surf zone area of a beach located by the end of “Rua Bolivia” in Guaratuba, Brazil (25^o ^55′ 47″ S, 48^o ^34′ 48″ W), and in “Praia das Gaivotas” located in Pontal do Paraná, Brazil (25^o ^43′ 07” S, 48^o ^28′ 49″ W) in March 2015 and January 2019 respectively. Specimens of *Zelinkaderes yong* were collected intertidally from fine sand, at Geumneung beach (33^o ^23′ 23″ N, 126^o ^14′ 04″ E) located at Jeju Island, South Korea in May 2018. The extraction of meiofauna in all sampling localities was done by mixing an equal part of seawater and sediment in a bucket, stirring the mix vigorously, letting the sediment settle and posteriorly filtering the supernatant through a 60 μm mesh. Kinorhynchs were sorted alive from the concentrated meiofauna sample.

### Light microscopy (LM)

Specimens of *C. styx* were fixed in 4 % paraformaldehyde, dehydrated through a graded series of water/glycerine, and incubated overnight in 100 % glycerine. Afterwards, individual specimens were mounted in Fluoromount G between two cover glasses, attached to an H-S plastic slide (Electron Microscopy Sciences, cat# 72,268) examined with an Olympus BX51 light microscope with differential interference contrast and imaged with an Olympus DP27 camera. Transmitted light images of *Z. yong* were acquired in a confocal laser scanning microscope (see details below) as a separate channel together with the fluorescent signal. *F. kalenesos´* light micrograph shown in Fig. 1B was kindly provided by Matteo Dal Zotto.

### Immunohistochemistry and confocal laser scanning microscopy (CLSM)

Specimens of *C. styx*, *F. kalenesos* and *Z. yong* were isolated, relaxed for 5-10 min in a MgCl_2_ solution, and fixed in 4 % paraformaldehyde (PFA) in filtered sea water for 40-50 min at room temperature. Posteriorly, the specimens were washed three times in phosphate buffered saline (PBS) and stored at 4^o^C in PBS with 0.05 % of Sodium azide (NaN_3_) to prevent microbial growth. Posteriorly, selected specimens of each species (18 *C. styx*, 20 *F. kalenesos* and 12 *Z. yong*) were used for immunohistochemical analyses. Prior to each experiment, the specimens were washed in PBS 1x and their cuticle was cut with a micro scalpel to facilitate the penetration of the stainings. Specimens were incubated overnight in blocking solution (PBT (PBS 1x + 0.25 % bovine serum albumin + 1 %Triton X-100)+ 6 % normal goat serum) and then treated with the following primary antibodies: polyclonal rabbit anti-serotonin (Sigma-Aldrich S5545), monoclonal mouse anti-acetylated α-tubulin (Sigma-Aldrich T6793), and polyclonal rabbit anti-FMRFamide (Immunostar 20,091) at a concentration of 1:400 in blocking solution at room temperature for 48 h. Afterwards, specimens were rinsed multiple times with PBT and immediately incubated at room temperature with the following secondary antibodies: sheep anti-rabbit IgG F (ab′)2 fragment labelled with Cy3 (Sigma-Aldrich C2306) and goat anti-mouse labelled with Cy5 (115-175-062; Jackson Immuno Research, West Grove, USA) in 1:400 concentration. After 48 h, the specimens were rinsed with multiple exchanges of PBT followed by three 15-min PBS rinses.

For CLSM imaging, specimens were individually mounted on glass slides in Vectashield® antifade mounting medium containing DAPI (VECTOR LABORATORIES, Burlingame, USA). Specimens were imaged using an Olympus IX 8 inverted microscope in combination with a FluoView FV1000 CLSM at the Biological Institute, University of Copenhagen; and an Olympus FV1000 Multiphoton CLSM at the UBC Bioimaging Facility. Z-stacks were analysed and composed with Fiji, version 2.0 (Wayne Rasband, National Institutes of Health) as well as Imaris v.7.2 (Bitplane). CLSM z-stack maximum projection images were edited with Adobe Photoshop CS6 (Adobe Systems Incorporated, San Jose, CA) and Fiji (e.g., rotation, contrast and brightness). Schematics and figure plates were arranged in Adobe Illustrator CS6.

Nervous system terminology follows the neuroanatomical glossary of Richter et al. [64]. Positional information used for the identification of external and internal anatomy, followed the commonly accepted terminology for kinorhynchs compiled in Sørensen & Pardos [65].

## Data Availability

The data underlying this article is available in the article, additional supporting material is available at the Electronic Research Data Archive (ERDA) of the University of Copenhagen 10.17894/ucph.8d8a3edb-5ba8-47d6-a26e-54f47347bb0b.
